# Analysis of Spiders' Joint Kinematics and Driving Modes under Different Ground Conditions

**DOI:** 10.1155/2019/4617212

**Published:** 2019-12-11

**Authors:** Xin Hao, Wenxing Ma, Chunbao Liu, Yilei Li, Zhihui Qian, Luquan Ren, Lei Ren

**Affiliations:** ^1^School of Mechanical and Aerospace Engineering, Jilin University, Changchun 130022, China; ^2^Key Laboratory of Bionic Engineering, Ministry of Education, Jilin University, Changchun 130022, China; ^3^School of Mechanical, Aerospace and Civil Engineering, University of Manchester, Manchester M13 9PL, UK

## Abstract

Although the hydraulic transmission system in spider legs is well known, the spider's mechanism of locomotion during different terrain conditions still need to be explored further. In this study, spider locomotion was observed in detail on three pavement test platforms: horizontal hard pavement, horizontal soft pavement, and sloped soft pavement. The movement characteristics and joint kinematics of *Grammostola rosea* legs were captured by high-speed cameras and Simi Motion 3D tracking software. These observations showed that the gait pattern was basically consistent with an alternating tetrapod gait; however, the pattern observed on the sloped soft pavement was slightly different from that of the two horizontal pavements. In particular, the duty factor of the spiders was 0.683 when walking on the horizontal hard pavement, 0.668 on the horizontal soft pavement, and 0.630 on the sloped soft pavement. The duty factor was greater than 60% in all three pavement environments, which was minimal when walking on the sloped soft pavement. This pattern showed that spiders might have superior stability when walking, but their stability decreased in the sloped soft pavement environment. The ranges of joint angles through the spiders' gait cycles in every pavement environment were also analysed and compared. The findings showed that the hydraulically driven femur-patella and tibia-metatarsal joint angles varied widely, which confirmed that hydraulically driven joints had major functions and obvious effects on the walking process. The kinematic patterns identified in this study provide improved understanding of the hydraulic transmission mechanisms, the factors that affect motion stability, and the design of biomimetic systems.

## 1. Introduction

After hundreds of millions of years of natural selection through survival of the fittest, organisms have evolved and their locomotion systems have developed in the direction of simplicity, reliability, efficiency, and adaptability [1]. Some organisms continue to evolve and optimise their motion systems in terms of physiology and morphology and improve their abilities for hunting, foraging, and escaping enemies [2]. These organisms have evolved special “biohydraulic systems.” For example, the starfish hydraulic system can achieve a variety of physiological motor functions [3]; the chafer uses hydraulic pressure to expand the hind wings [4]. They are all able to achieve efficient drive and motion while maintaining low internal pressures. Such systems are compact, pollution-free, efficient, and reliable.

Spiders are also a typical example of creatures with “biohydraulic systems.” These animals have high-efficiency hydraulic systems in their legs, which allow them to achieve rapid movement for capturing prey [5]. The spider has a total of eight legs (aside from the first pair of tentacles), and these legs are composed of seven sections: the coxa, trochanter, femur, patella, tibia, metatarsus, and tarsus [6]. The tibia-metatarsus joint and the femur-patella joint have been reported as pure hydraulic joints. These joints have no extensor muscles [7], and their hydraulic driving force generates torque, so that the joint connections can extend backwards [8].

Previous studies have been conducted on the kinematic mechanisms for spiders walking. Since 1985, Anderson et al. have explored the effects of different spider movements in terms of physiology and energy efficiency [9–11]. Also, Shultz and Ward and Humphreys compared differences in the motion mechanisms of *Lycosa rabida Walckenaer*, *Dolomedes triton*, *Trochosa ruricola*, and *Lycosa tarantula* [12, 13]. Wilson described the tarantula's gait in terms of the phase relationships between leg pairs and found that the variations of different stepping sequences are basically independent of speed [14]. Spagna and Mohan analysed the gait characteristics of two fast-moving spider species (including the characteristics of an aerial phase) and revealed how these spiders achieved their significant speed [15]. Roberts et al. investigated the walking kinematics of Pycnogonida and found out the extremely slow walking speeds and variable gait patterns of sea spiders compared to those of terrestrial spiders [16]. Biancardi et al. determined the velocity boundary (11 cm/s) and the differing characteristics of the two main gaits used by *Grammostola mollicoma*. This study involved analysis of several variables, such as stride length and frequency, duty factor, mechanical external work, and energy recovery [17]. Zeng and Crews explored biomechanics of omnidirectional strikes in spiders by the leg orientation, gait configuration, linear velocity, rotational velocity, and acceleration of Selenopidae based on translational and rotational movements [18]. Wang et al. used a 3D locomotion observation system to record the movements of spiders' legs, the shifts in their centre of mass, and the changes in their joint rotation angles [19]. Booster et al. explored the effects of temperature on the leg kinematics of sprinting tarantulas by measuring the coefficients of two hydraulic joint angles. They found that high-speed motion could constrict the hydraulic joints [20].

Recently, a number of researchers have conducted experiments towards developing spider-like hexapod robots, and some bionic flexible drive mechanisms have been inspired by the hydraulic joints of spiders. For example, Carlo Menon and Cristian Lira designed a driving structure known as a “Smart Stick,” which is modelled on a bionic spider joint and uses a flexible hydraulic actuator [21]. Landkammer et al. designed a hydraulically driven system that extended through an increase in fluidic pressure (unlike flexion, which is performed by muscles) [22]. However, the mechanism by which hydraulic drives operate still needs further study. Likewise, its hydraulic drive mechanism is still not fully resolved. For example, the current research lacks the kinematics of spiders on multiple pavements and the comparison of hydraulic joint angles with other joints, which is helpful to clarify their working mechanisms.

Here, we proposed two primary hypotheses for spiders' joint kinematics and driving modes under different ground conditions: (1) The more complex the pavement environment is, the lower stability the spider has when walking, and the gait pattern may even change. (2) Hydraulically driven joints have obvious effects on the walking process, and the joint angles vary more widely than other joints. In this study, *Grammostola rosea* tarantulas were determined as the subject, and their gait pattern and joint kinematics were analysed under three different terrain conditions, including horizontal hard pavement, horizontal soft pavement, and sloped soft pavement. The findings may help to clarify the operating principles governing the movement of spider legs under different surface and angle conditions. The changes in the angles of the hydraulic joints were also compared with that of ordinary pure muscle joints.

## 2. Materials and Methods

### 2.1. Animals

**Figure 1:**
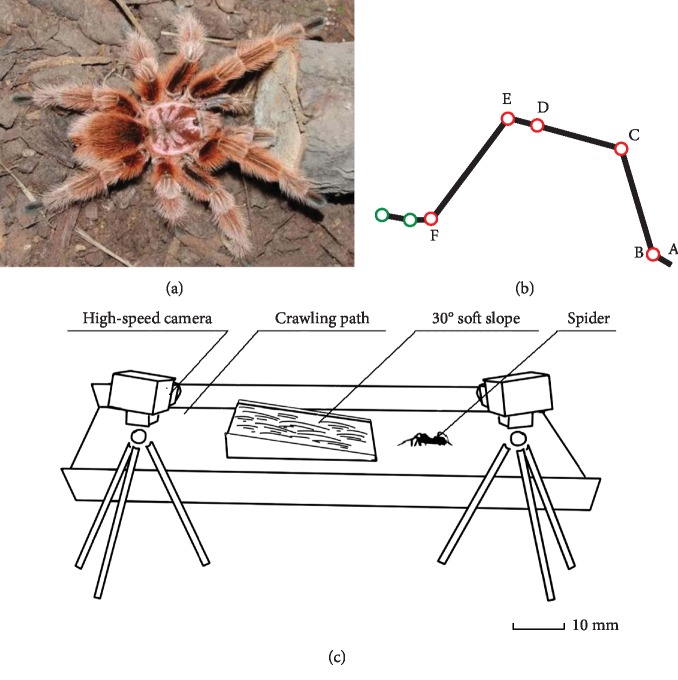
Experimental sample and diagram of experimental system.

All measurements in this study were made on adult specimens of *Grammostola rosea*, as shown in Figure 1(a). *Grammostola rosea* belongs to the phylum Arthropoda, integral subphyla Arachnida, Araneae, Protoarachnidae, Scarachnidae, and Avian Araneae [23]. Three *Grammostola rosea* spiders, weighing between 30 g and 34 g, with body lengths ranging from 60 mm to 80 mm, were selected for this experiment. The information for each spider used in the study was listed in Table 1.

Six reflective markers were placed at the five joints (B, C, D, E, and F) of each spider leg and at the tip (A) of the claw, respectively. There were 24 markers on the left four legs. Then, four joint angles were defined (see Figure 1(b)). Angle ABC was the metatarsus-tarsus joint angle, angle BCD was the tibia-metatarsus joint angle, angle CDE was the patella-tibia joint angle, and angle DEF was the femur-patella joint angle. No markings or measurements of the coxa or the trochanter were made in this experiment due to the short lengths of these structures in the spiders' legs, the difficulty of observing and tracing their positions, and the small range of angle changes made by these structures during movements.

### 2.2. Measurements

The experimental system was composed of a pair of high-speed cameras (448 × 336 pixels, 240 fps; EX-FH25, Casio, Tokyo, Japan) and a runway. The runways were of three types: horizontal hard pavement, horizontal soft pavement, and 30-degree sloped soft pavement as shown in Figure 1(c). Before the experiment began, a measuring space of 0.6 m × 0.4 m × 0.2 m was calibrated with a 3D calibration frame. The two high-speed cameras formed a motion-tracking system with an average error of ±1.0 mm that recorded videos of the spiders walking freely through the runway. Twenty trails were repeated for each of the three pavement environments, and a total of 37 sets of video data of the spiders that did not stop midway and deviated from the runway during the walking movement were selected and saved. Simi Motion (Simi Reality Motion Systems, Unterschleißheim, Germany), a 3D motion analysis system, was used to track and test the 3D coordinates of the 24 marker points and joint parameters. The number of frames in a gait cycle was tracked, and a series of gait parameters was calculated.

## 3. Results and Discussion

### 3.1. Gait Characteristic Parameters

In this study, a complete gait cycle was defined as starting when the first leg on the spider's left side touched the ground and ended the next time that this leg touched the ground. During an entire cycle, all eight legs went through stance phase and swing phase.

In denoting these leg movements, L and R indicate left and right, and the numbers start from the first pair of feet in the anterior-posterior sequence. Therefore, L_1_ stands for the first leg on the left and R_1_ stands for the first leg on the right. The eight legs are denoted as L_1_, L_2_, L_3_, L_4_, R_1_, R_2_, R_3_, and R_4_, respectively. As the structure of *Grammostola rosea* is symmetrical, the important parameters of the left leg and the right leg are not significantly different, so the data on both sets can be combined for analysis [19]. Twenty experiments were carried out for each of the three types of pavements, and twelve sets of valid experimental data were selected for analysis. The important parameters obtained are shown in Table 2. The duty factor is the time taken up by the support phase compared to that for the entire cycle [24].

Table 2 shows that when the *Grammostola rosea* spiders walked on the three types of pavements, the duty factor of each leg was between 60% and 75%. The duty factor on the horizontal hard pavement was slightly larger than that on the soft pavement, and the duty factor on the sloped soft pavement was much smaller than that on the two horizontal pavements. These findings showed that when the spiders walked normally, all eight legs spent much more time in a ground support position than in an air swing position during each complete gait cycle. This preponderance of the ground support position gave spiders better stability in walking. When walking on a soft pavement, the spiders altered the proportion of stance phase and swing phase movements, to adapt to the pavement's condition. On the sloped soft pavement, the spiders showed less stability than when walking on the other two pavements.

The spiders walked slower on the soft pavement, and the distance covered by each leg step was smaller. The velocity and the single-leg step distance were lowest when the spiders were walking on the sloped soft pavement, but the time needed for each complete cycle was the longest.

### 3.2. Gait Pattern

**Figure 2:**
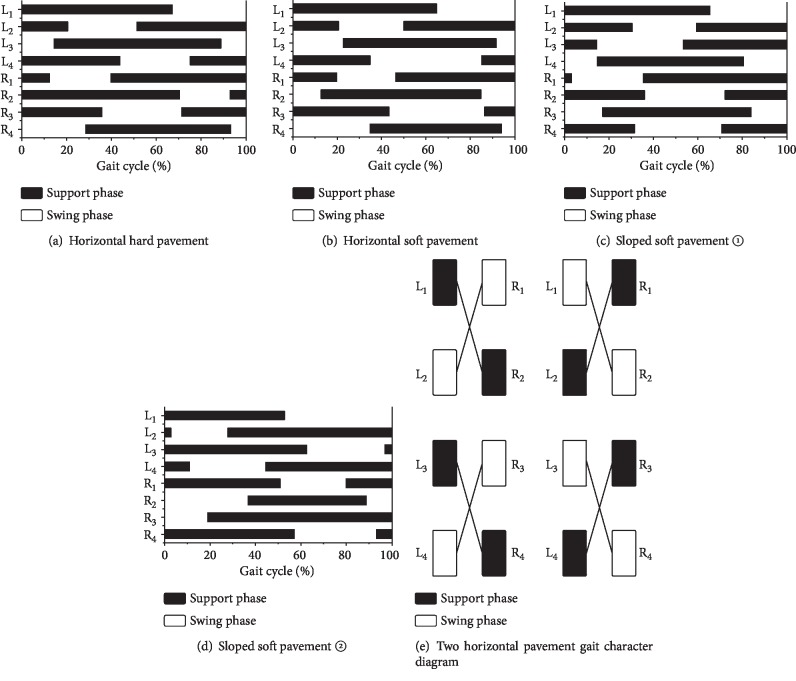
Gait pattern diagrams.

Figures 2(a)–2(d) show a series of gait diagrams, illustrating the footfall patterns within each gait cycle for spiders walking on the three types of pavement. As the gait on the sloped soft pavement was the most complicated, two of the differing gait patterns observed on the sloped soft pavement were selected for analysis.  Figure 2(e) shows gait diagrams of the spiders' walking patterns on the two horizontal pavements, in which the black bars indicate the support phase and the white sections indicate the swing phase.

As could be seen in Figure 2, the spiders had at least five legs on the ground at all times when walking on the two horizontal pavements. Most of the time, six legs were on the ground, which helped to ensure stability. When walking on the sloped soft pavement, the spiders had only four or five legs on the ground over most of the gait cycle, so their stability may be less than when walking on horizontal pavements.

In comparing the gait diagrams of spiders walking on horizontal pavements, it could be seen that the fourth pair of legs had a larger swing amplitude and a longer swing phase when walking on soft pavement than when walking on hard pavement. This pattern may represent the fact that the fourth pair of legs is longer than the others. During the support period, the force on these longer legs was larger, and the claw tips were pressed into the soil, so their swing periods were longer.

When walking on the two horizontal pavements, the legs showed the following patterns of regularity. First, along each side of the spider, the motions of every second leg were basically the same; that is, L_1_ and L_3_ moved together, as did L_2_ with L_4_, R_1_ with R_3_, and R_2_ with R_4_. Second, the motion states of each diagonal pair of legs were basically the same; that is, L_1_ and R_2_ moved together, as did L_2_ with R_1_, L_3_ with R_4_, and L_4_ with R_3_. Third, on each side of the spider, the motion states of each adjacent leg were different; that is, when L_1_ was supporting, L_2_ was swinging, and when L_3_ was swinging, L_4_ was supporting. Fourth, the motion states of each diagonal pair of legs on opposite sides of the spider were different; that is, when L_1_-R_2_ and L_3_-R_4_ were swinging, R_1_-L_2_ and R_3_-L_4_ were supporting. This pattern of motion is called an alternating tetrapod gait, and it has fairly good stability. The stepping sequence of the spiders could be 4-2-3-1, 2-3-1-4, 3-1-4-2, or 1-4-2-3, because each leg could start the cycle [25]. If any pair among the four pairs of legs was ignored, the gait would switch to a triangle gait, as is displayed by many arthropods. The stepping patterns in a tripod gait are L_1_-R_2_-L_3_ and R_1_-L_2_-R_3_.

The two gait patterns that the spiders most commonly used when walking on the sloped soft pavement were the following: First, only the legs on one side moved together, as was consistent with an alternating gait—either the right four legs (Figure 2(c) or the left four legs (Figure 2(d)). Second, the middle two legs of each side basically moved together (the second and third legs on the left side of Figure 2(c), and the second and third legs on the right side of Figure 2(d)).

### 3.3. Joint Angle Variation

**Figure 3:**
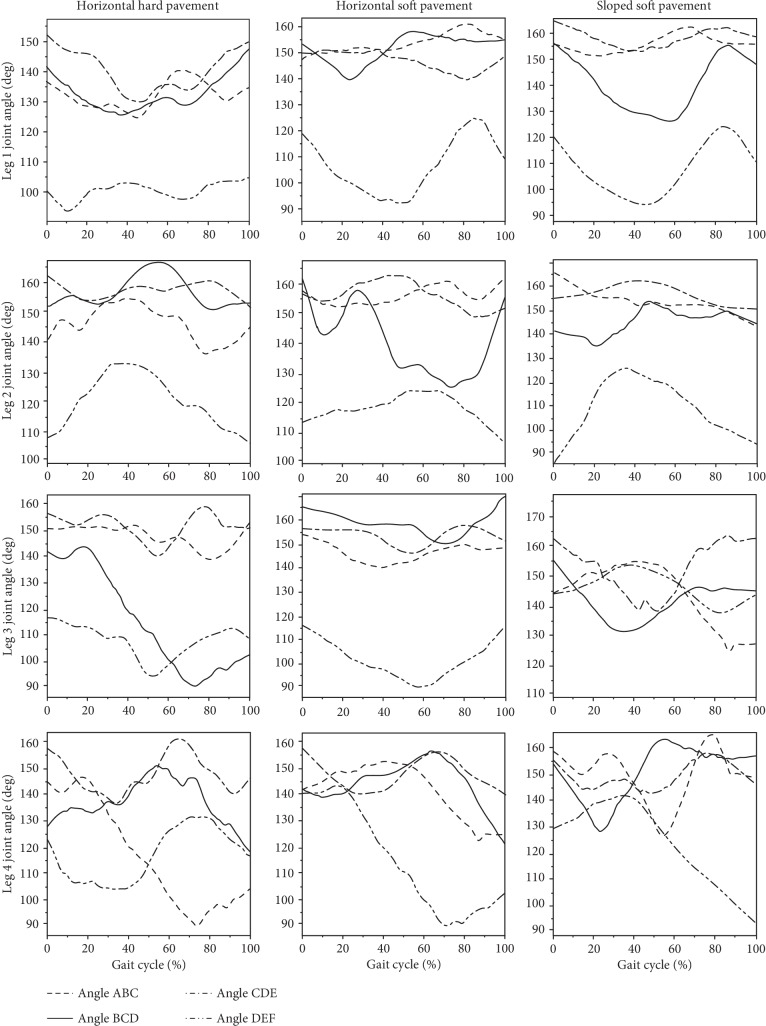
Variations of joint rotation angles during the whole gait cycle.

Five groups of data pairs (of joint angle and time measurements) were derived for each of the three pavement conditions. The normalisation method was adopted to solve the problem that the spiders had different speeds and different gait cycle times during each test.  Table 3 shows the extreme values and the ranges (means ± s.d.) in the rotation angles of the spiders' leg joints.  Figure 3 illustrates the relationships between the mean of joint angles of each leg in each gait cycle, under each of the three pavement conditions.

Leg 1 played the role of guiding, exploring, and buffering during walking [19]. Within each gait cycle, the range of the joint angle ABC was smaller when walking on the sloped soft pavement than it was when walking on the two horizontal pavements. The range of the joint angle BCD was significantly larger when walking on the sloped soft pavement than when walking on the two horizontal pavements. On the horizontal hard pavement, the joint angles CDE and DEF were significantly larger than they were on the two soft pavements.

Leg 2 helped to maintain the lateral stability of movement and assisted in support during walking [19]. For this leg, the horizontal soft pavement had the greatest effect on the range of joint angle changes within each gait cycle. The range of joint angles for ABC when walking on horizontal hard pavement was significantly larger than that on the two soft pavements. On the horizontal soft pavement, the range of joint angle BCD was much larger than that seen on the other two types of pavement. Overall, the range of joint angle CDE was the largest, and the range of joint angle DEF was the smallest.

The function of Leg 3 was basically the same as that of Leg 2 [19]. However, within each gait cycle, the joint angle ABC had the largest range when walking on the sloped soft pavement, and the joint angle DEF had the smallest range. When the spiders were walking on horizontal hard pavement, the ranges of joint angles BCD and CDE were obviously larger than when walking on the two soft pavements.

Leg 4 served as a major driving force for pushing the body forward [19]. For this leg, the horizontal soft pavement had the greatest impact on the range of joint changes within each gait cycle. The joint angles ABC, BCD, and DEF had the largest range of movement when walking on horizontal soft pavement, and the joint angle CDE had the smallest range.

For all legs, the femur-patella joint (DEF) angle had the smallest range of variation of roughly between 130° and 90°. The other three angles generally varied between 170° and 140°. The hydraulically driven joints (the tibia-metatarsus (BCD) and the femur-patella joint (DEF)) had the largest range of joint angles and the steepest slopes. To a certain extent, these findings showed that the hydraulically driven joints played a greater functional role in walking.

By combining the diagrams of joint angle changes (in Figure 3) with the diagrams of gait patterns (in Figure 2), it could be seen that within each complete gait cycle, the changes for Leg 1 in each of the three pavement conditions were similar. The phase pattern changed from support to swing. For Leg 2, the gait changed similarly from a support to swing phase pattern. The changes in the joint angle appeared as a curve with one peak and two troughs. In the two horizontal pavement conditions, Leg 3 displayed a swing-support-swing phase pattern. The changes in joint angle appeared as two peaks and one trough. Leg 4 showed a support-swing-support phase pattern, and the joint angle change curve showed one peak and two troughs. In the sloped soft pavement condition, when the Leg 3 gait changed to the support-swing-support phase, the curves for the joint angles ABC and CDE showed two peaks and two troughs, and the joint angles BCD and DEF showed one peak and one trough. When Leg 4 changed to the swing-support-swing phase, the curve for the joint angle change showed only one peak and one trough.

Concerning the two hydraulic joints, the tibia-metatarsus joint (BCD) had roughly the same trends and ranges of change in both Leg 1 and Leg 2. In Leg 3 and Leg 4, the joint angles had the same trends when walking on the two horizontal pavements, but they showed opposite trends when walking on the sloped soft pavement. On the two soft pavements, the femur-patella joint (DEF) in Leg 1 showed roughly the same trends in both the angles and the phase changes, but it showed opposite trends when walking on the hard pavement, and the amplitude of these shifts was smaller. In Leg 2, the joint angles had the same trends with all three types of pavements, but these joints showed greater phase changes when walking on the sloped soft pavement. In Leg 3, the angles had the same trends when walking on the two horizontal pavements, but they had opposite trends when walking on the sloped soft pavement. For Leg 4, the joint angles were almost the same on each of the three pavements, but they showed an opposite trend when walking on the hard pavement. It could be seen that the sloped pavement had a greater influence on the tibia-metatarsus (BCD) joints of Leg 3 and Leg 4. The hard pavement had a greater impact on the femur-patella (DEF) joints of Leg 1 and Leg 4. Also, the sloped soft pavement had a greater impact on the DEF joints of Leg 2 and Leg 3. These data may provide support for further analysis of spider hydraulic walking mechanisms and for the future bioinspired design of spider-like hydraulic robots.

Due to the complexity of the research object and the limitations of the experimental conditions, only three experimental samples were selected in this study, which might lead to the potential limitations of the experimental approach. Small sample size means smaller power. Studies may provide false-positive results and false-negative results, which cause subsequent studies to build upon the incorrect results or for potentially important findings to go undetected [26]. We conducted more experiments on each spider and obtained more experimental data. We will conduct a larger sample of research in the future.

## 4. Conclusions

Differences were detected in the gait parameters of spiders walking in three differing environments. The duty factor for the spiders' eight legs (that is, the support period) was greater than 50% on all three pavement environments. The duty factor was largest when the spiders were walking on the sloped soft pavement, second greatest when walking on the horizontal soft pavement, and smallest when walking on the horizontal hard pavement. This pattern showed that the spiders had good stability when walking, but their stability may become lower in the sloped soft pavement environment. The horizontal hard pavement allowed the highest speed with the shortest cycle time, and the sloped soft pavement caused the lowest speed and the longest cycle time. The gait pattern was found basically consistent with the alternating tetrapod gait, but the gait pattern observed on the sloped soft pavement differed slightly from that observed on the two horizontal pavements. The hydraulically driven femur-patella (DEF) and tibia-metatarsus (BCD) joints showed widely varying angles, which indicated that hydraulically driven joints have a major role, and have more obvious effects in spider walking than the other joints. These data could provide support for further analyses of spider hydraulic walking mechanisms, motion stability control, and the design of bionic hydraulic robots.

Some problems addressed in this study require further research and discussion. To further investigate the dynamics of spider movement, a special test system is needed to analyse the changing factors affecting the ground contact forces that spiders exert in different ground conditions. Such analysis will allow greater understanding regarding the role of force states in motion, and of the ways that spiders exert control to ensure both stability of motion and stability of the lateral force. The coordinated control mechanism used in the process of exercise needs to be clarified. It should also be possible to combine dynamics with kinematics to constrain spatial positions, in accordance with kinematic information on factors such as motion gait and joint rotation angle. Such analysis may make it possible to generate internal driving force and hydraulic transmission, with consideration of the mechanical properties of the foot material. A theoretical calculation method could be also used to derive the driving principles of the foot and the processes of bioenergy transmission and transformation. Further investigation along these lines can reveal the mechanisms by which spider hydraulic systems use biohydraulic energy to drive themselves efficiently.

## Figures and Tables

**Table 1:** Spider information.

	Weight (g)	Body length (mm)	Leg length (mm)			
			Leg 1	Leg 2	Leg 3	Leg 4
Spider 1	31.42	62.5	62.5	53.7	52.6	58.9
Spider 2	34.58	75.4	57.2	43.8	52.0	58.4
Spider 3	32.46	63.7	61.5	53.8	46.8	55.9

**Table 2:** Gait parameters on three pavements.

Parameters	Leg	Horizontal hard pavement	Horizontal soft pavement	Sloped soft pavement
Average velocity (m/s)		0.11 ± 0.015	0.08 ± 0.022	0.06 ± 0.016

Step distance (mm)	1	33.5 ± 2.6	27.5 ± 3.1	26.0 ± 2.1
	2	31.5 ± 2.2	30.5 ± 2.1	28.0 ± 2.8
	3	37.5 ± 3.0	25.0 ± 2.6	28.0 ± 3.6
	4	29.0 ± 2.0	28.5 ± 2.4	28.5 ± 2.5

Gait cycle (s)		1.55 ± 0.28	1.64 ± 0.31	1.77 ± 0.21

Duty factor	1	0.68 ± 0.03	0.69 ± 0.07	0.65 ± 0.05
	2	0.69 ± 0.06	0.71 ± 0.02	0.64 ± 0.09
	3	0.70 ± 0.05	0.67 ± 0.10	0.61 ± 0.10
	4	0.66 ± 0.03	0.60 ± 0.08	0.62 ± 0.05

Note: the values in the table are means ± s.d..

**Table 3:** Extreme values and ranges of rotation angles of the spiders' leg joints (°).

		Horizontal hard pavement				Horizontal soft pavement				Sloped soft pavement			
		1	2	3	4	1	2	3	4	1	2	3	4
ABC	Max	162.7 ± 12.3	155.7 ± 9.7	152.1 ± 27.4	102.6 ± 25.6	162.5 ± 9.0	162.8 ± 13.5	154.1 ± 17.2	156.6 ± 3.7	161.4 ± 8.3	165.4 ± 13.1	161.5 ± 9.4	152.4 ± 5.2
	Min	146.4 ± 6.0	136.5 ± 17.1	138.7 ± 22.7	88.5 ± 2.4	148.4 ± 11.9	152.5 ± 10.0	142.5 ± 27.2	128.3 ± 17.2	152.6 ± 12.6	145.4 ± 19.6	142.6 ± 9.1	126.5 ± 17.1
	Range	16.0 ± 13.7	19.1 ± 19.7	13.4 ± 35.6	14.1 ± 25.8	14.1 ± 15.0	10.3 ± 16.8	11.6 ± 32.2	28.3 ± 17.6	8.8 ± 15.1	20.0 ± 23.5	18.9 ± 13.1	25.9 ± 17.8

BCD	Max	165.7 ± 9.8	166.2 ± 15.5	143.4 ± 4.4	152.3 ± 7.2	159.3 ± 8.8	162.1 ± 14.2	173.4 ± 9.3	157.3 ± 16.9	156.6 ± 12.1	152.4 ± 14.9	158.6 ± 8.8	155.1 ± 11.3
	Min	147.6 ± 6.8	150.5 ± 17.2	90.5 ± 1.1	123.4 ± 7.0	141.4 ± 9.1	124.6 ± 12.8	150.2 ± 19.1	120.2 ± 11.2	130.4 ± 3.0	136.6 ± 5.6	140.4 ± 8.2	130.2 ± 8.6
	Range	18.1 ± 12.0	15.7 ± 23.1	52.9 ± 4.6	28.9 ± 10.1	17.9 ± 12.6	37.5 ± 19.1	23.2 ± 21.2	37.1 ± 20.2	26.2 ± 12.5	15.8 ± 15.9	18.2 ± 12.7	24.9 ± 14.2

CDE	Max	173.1 ± 4.5	162.6 ± 13.0	160.9 ± 6.5	162.5 ± 16.8	151.2 ± 7.0	164.6 ± 13.9	156.4 ± 11.1	158.6 ± 22.3	165.2 ± 2.6	162.3 ± 14.6	155.3 ± 10.1	162.4 ± 5.1
	Min	150.6 ± 8.9	149.3 ± 22.4	141.2 ± 9.4	136.8 ± 11.1	140.2 ± 10.4	148.3 ± 129	148.2 ± 7.8	139.8 ± 6.5	153.2 ± 6.1	150.4 ± 10.3	148.3 ± 15.2	131.8 ± 13.4
	Range	22.5 ± 10.0	13.3 ± 25.9	19.7 ± 11.5	25.7 ± 20.1	11.0 ± 12.5	16.3 ± 19.0	8.2 ± 13.6	18.8 ± 23.2	12.0 ± 6.6	11.9 ± 17.9	7.0 ± 18.2	30.6 ± 14.4

DEF	Max	121.5 ± 14.6	135.5 ± 17	116.4 ± 18.9	131.6 ± 19.3	125.1 ± 0.3	125.7 ± 13.2	116.5 ± 2.1	145.2 ± 8.9	125.3 ± 1.2	124.5 ± 5.9	142.7 ± 4.7	153.8 ± 12.6
	Min	83.5 ± 14.4	108.3 ± 13.9	95.3 ± 9.6	102.5 ± 18.1	95.8 ± 2.3	108.3 ± 5.5	94.6 ± 15.0	92.6 ± 7.1	94.7 ± 2.2	84.9 ± 12.1	91.8 ± 7.0	141.5 ± 18.3
	Range	38.0 ± 21.3	27.5 ± 14.0	21.1 ± 21.2	19.1 ± 26.4	29.3 ± 2.4	17.4 ± 14.3	21.9 ± 15.1	52.6 ± 11.3	30.6 ± 2.5	39.6 ± 13.5	50.9 ± 8.4	12.3 ± 22.2

## Data Availability

The data used to support the findings of this study are available from the corresponding author upon request.
